# Malnutrition in Chakradharpur, Jharkhand: an anthropological study of perceptions and care practices from India

**DOI:** 10.1186/s40795-019-0299-2

**Published:** 2019-07-02

**Authors:** Ipsha Chaand, Minashree Horo, Mohit Nair, Amit Harshana, Raman Mahajan, Vivek Kashyap, Fernanda Falero, Montse Escruela, Sakib Burza, Rajib Dasgupta

**Affiliations:** 1 Jawaharlal Nehru University, New Delhi, India; 2 Medecins Sans Frontieres, New Delhi, India; 3 Rajendra Institute of Medical Sciences, Ranchi, India; 4 Medecins Sans Frontieres, Barcelona, Spain

**Keywords:** Malnutrition, Qualitative, CMAM

## Abstract

**Background:**

This study aims to investigate the knowledge, perception and practices related to health, nutrition, care practices, and their effect on nutrition health-seeking behaviour.

**Methods:**

In order to have maximum representation, we divided Chakradharpur block in Jharkhand state into three zones (north, south and centre regions) and purposively selected 2 Ambulatory Therapeutic Feeding Centre (ATFC) clusters from each zone, along with 2 villages per ATFC (12 villages from 6 ATFCs in total). In-depth interviews and natural group discussions were conducted with mothers/caregivers, frontline health workers (FHWs), Medicins Sans Frontieres (MSF) staff, community representatives, and social leaders from selected villages.

**Results:**

We found that the community demonstrates a strong dependence on traditional and cultural practices for health care and nutrition for newborns, infants and young children. Furthermore, the community relies on alternative systems of medicine for treatment of childhood illnesses such as malnutrition. The study indicated that there was limited access to and utilization of local health services by the community. Lack of adequate social safety nets, limited livelihood opportunities, inadequate child care support and care, and seasonal male migration leave mothers and caregivers vulnerable and limit proper child care and feeding practices. With respect to continuum of care, services linking care across households to facilities are fragmented. Limited knowledge of child nutrition amongst mothers and caregivers as well as fragmented service provision contribute to the limited utilization of local health services. Government FHWs and MSF field staff do not have a robust understanding of screening methods, referral pathways, and counselling. Additionally, collaboration between MSF and FHWs regarding cases treated at the ATFC is lacking, disrupting the follow up process with discharged cases in the community.

**Conclusions:**

For caregivers, there is a need to focus on capacity building in the area of child nutrition and health care provision post-discharge. It is also recommended that children identified as having moderate acute malnutrition be supported to prevent them from slipping into severe acute malnutrition, even if they do not qualify for admission at ATFCs. Community education and engagement are critical components of a successful CMAM program.

## Background

Child undernutrition continues to occupy a significant share of the global health burden: in total, around 161 million children under-five (U5) are chronically malnourished, 99 million children are underweight, and 45% of child deaths are attributable to malnutrition [1]. Despite robust economic growth, India is home to 40% of the world’s malnourished children and 35% of low birth weight infants in developing nations [2]. Indian states with chronic poverty and chronic hunger exhibit both severe acute malnutrition (SAM) as well as severe chronic malnutrition (SCM) (i.e. severe wasting and severe stunting) [3].

Chronic malnutrition in India is largely attributed to poor complementary feeding, especially during the first year of life [2], intergenerational malnutrition [4, 5], poor or limited ante-natal care (ANC), poor immunization coverage, high work load on mothers, and lack of state intervention in child care [6].

Jharkhand experiences high levels of multi-dimensional poverty with 88% of the population classified as poor, according to the multi-dimensional poverty index (MPI). The state also had a relatively slow reduction of poverty and a low monthly per capita expenditure (MPCE)^1^ between 2004 and 2012. The incidence of rural poverty in the state decreased from 65.5 to 42.9% during this period, but continues to be substantially higher than the national average [7, 8]. The state fares poorly on both the Hunger Index (ranked 16 out of the 17 major states) and undernutrition indicators: more than one in every four children are severely underweight and about one in three are wasted. These indicators are even worse among tribal communities within the state.

Previous studies have explored the seasonal patterns and cycles through which the chronically poor acquire food and uncovered complex mechanisms adopted by tribal households during periods of food insecurity for managing food supplies. Safety-net programs also fall short of need-based expectations and fail to achieve desired impacts, particularly in the context of women and children [9].

A recent study conducted in the state highlighted the unsatisfactory performance of the Integrated Child Development Scheme (ICDS) and finds a mismatch between poor demand for ICDS services and irregular supply of the offered services from the system [10]. Despite repeated emphasis on management of under-nutrition among under-5 children, existing health services are yet to respond to this challenge in any significant manner [11]. Infant and young child feeding (IYCF) practices are highly inadequate and age inappropriate; these include aspects of breast feeding, complementary feeding, feeding during illness, and discordance between mothers’ knowledge and practices [12]. The state witnessed a 13% decline in adequate infant and child feeding from the National Family Health Survey (NFHS) 3 to NFHS 4 [13].

Nutrition rehabilitation models in India - through Malnutrition Treatment Centres (MTC) or Community-based Management of Acute Malnutrition (CMAM) - currently seek to treat Severe Acute Malnutrition (SAM). Evidence indicates that the incidence of default from these treatment programmes and relapse rates in malnourished children are high; understanding the drivers behind this will be critical in ensuring a successful community-based service [14–16].

The specific objectives of this study were to:

- understand community perceptions of malnutrition and its effect on nutrition related health-seeking behaviour.
- investigate causes and determinants of malnutrition.
- examine social, economic and cultural structures at the community level and their implications on continuum of care (CoC).
- provide operational recommendations to guide the development of community interventions in relation to CMAM programs, health promotion, and prevention of malnutrition.

## Methods

### Study location

We purposively selected Chakradharpur block in West Singhbhum district due to the presence of functional ambulatory therapeutic feeding centres (ATFCs). The Medecins Sans Frontieres (MSF) program in Chakradharpur block is based on a CMAM model: a standard CMAM program consists of treatment sites (ATFCs) close to the community where children with uncomplicated SAM, who constitute the majority of patients (70–85%), can be seen on a weekly basis. SAM children with associated medical complications which require specialized medical attention are admitted to an inpatient facility known as a Malnutrition Treatment Center (MTC). The MSF CMAM program includes a cluster of 30 ATFCs distributed all over Chakradharpur block. ATFCs are located close to the communities they serve, and each ATFC cluster covers approximately 5–6 villages.

The total block population is 197,953, with 18 panchayats and 183 villages (169 inhabited and 14 un-inhabited) according to Census data from 2011. The block is predominantly populated with tribal communities (62%), with a relatively small Scheduled Caste population (approximately 5%). The tribal population is predominantly comprised of Ho tribes and the main language is Ho, along with Santhali and Mundari.

West Singhbhum has four functional MTCs. MTC Chakardharpur (located at the Block Health Center) and MTC Chaibasa (located at the District Hospital) were purposively selected to map the patient flow of SAM cases from the selected block.

### Study design

We adopted a maximum variation sampling approach to purposefully sample for population heterogeneity and maximum representation. We divided the block into three zones representing the north and south (hilly areas with thick forest cover) and central regions (plain/valley areas).

All villages are a mix of tribal and non-tribal households. We selected two ATFC clusters from each of the zones. Two villages were purposively selected based on their location: one village was located closest to the ATFC and the other farthest from the ATFC. Twelve villages from 6 ATFC clusters were selected (Table 1); 6 villages had maximum saturation of ATFC services and 6 villages were unserved by any ATFC.

**Table 1:** Village selection

Zone	ATFC	Villages
North	Kamegera	Harjoda, Hoyohatu
	Bharnia	Hatnabera, Dhukri
Central	Chainpur	Chandri, Dhutkundi
	Roladih	Sarjomhatu, Hathnatodang
South	Baipi	Bordiri, Boiysai
	Tuiya	Sagipi, Sirkapi

Two categories of mothers/caregivers were selected for this study:

- Mothers/caregivers accompanying children to the MTC (admitted in the MTC and completed a minimum stay of 7 days), in order to understand institutional care issues.
- Mothers (of children aged 6 months to 5 years) from the selected villages who are available and willing to participate, in order to understand community and village level issues.

We also purposively selected frontline health workers (FHWs), such as angwanwadi workers (AWW), auxiliary nurse midwives (ANMs), and accredited social health activists (ASHAs, interchangeably known as Sahiyas). The study also included MSF staff, nursing staff at MTCs, health educators, nutritional link workers (NLWs), nursing staff working at the selected ATFCs, and community representatives from Panchayati Raj Institutions (PRI) as well as village leaders (known locally as Mundas).^2^

The study was conducted in two phases. The first phase of data collection occurred over a period of 4 months, wherein the team conducted 5 natural group discussions (NGDs) with tribal mothers and 2 NGDs with non-tribal mothers across the three zones. Additionally, the team conducted in-depth interviews with 23 FHWs (5 AWWs, 13 Sahiyas, and 5 ANMs), and 7 community representatives (Table 2).

**Table 2:** In-depth interviews -- Respodent catgories and numbers

Respondent category	Number of respondents
Mothers	29
Accredited Social Health Activist/Sahiya	13
Auxiliry Nurse Midwife	5
Anganwadi workers	5
Community representatives	7

Following this initial phase, the team translated the data from Ho, Santhali and Hindi into English and conducted a preliminary analysis. The data was back-translated for quality assurance. Three members of the research team read each of the transcripts, and generated codes for themes emerging from the data. Data was inductively and deductively analysed, as some themes were pre-determined from the literature review while others emerged organically. Data was analysed separately for different stakeholders and reanalysed to assess similarities and differences in perceptions and practices across stakeholders. A final coding framework emerged by consensus among the three researchers.

Following this phase of analysis, researchers conducted further fieldwork for non-formal, unstructured interviews with all stakeholders in order to triangulate findings and conduct respondent validation (Table 3). Detailed field notes were prepared and some additional perspectives emerged; the interactions continued until saturation was attained.

**Table 3:** Respondent categories and numbers for non-formal interviews

Respondents for non-formal interactions	Number of participants
Mothers	35 (10 mothers from 6 villages, 14 mothers at ATFCs, 11 mothers at MTCs)
Doctors (MSF)	4
MSF program staff	29

Separate, semi-structured interview guides were prepared for the various stakeholder groups. The tools were prepared in English, and subsequently translated and back-translated from English to Hindi for quality assurance purposes. Data collection and analysis took place concurrently; hence, quality assurance was integrated directly into the approach through course corrections. The team took great caution to translate and transcribe audio recordings, including all words, phrases and pauses in conversation, as accurately as possible. The final data was triangulated through transcripts from unstructured interviews and facility observations using checklists in 8 different ATFC units, MTC Chakradharpur, as well as MTC Chaibasa.

## Results

### Perceptions and practices regarding malnutrition at the community level

#### Preference for home births is linked to initiation of breastfeeding

Home births are preferred by mothers over institutional delivery. Decisions regarding institutional births are made on the basis of distance, time (hour of the day), availability of monetary resources, availability of transport, duration of pre-labour, availability of health care personnel accompanying the women, and previous care experiences.

“It is more feasible for us to give birth at home because at least we manage the basic things at home. For example, like now (winter season), if we go to the hospitals for the child’s delivery, there is no proper facility. The hospital room or ward becomes so cold that it is very difficult to stay there. At least we manage to get fire wood that we use to keep the room warm where the mothers give birth. This is why most of us do not prefer to go to the hospitals, but instead choose birthing at home with the help of *dai* (traditional midwife) and elderly women.” [Mother, tribal]

Women who gave birth at an institution reported to have initiated breastfeeding within an hour of birthing. In contrast, the majority of women who birthed their babies at home reported intiation of breastfeeding between 2 and 3 h post-delivery, which occasionally extended till the next day.

“I breastfed my baby for the first time 2-3 hours after birth. I fed the baby only after cleaning was completed by the dai at home; it takes time for the cleaning process to finish. And then I cleansed my nipples and threw a few drops of the thick yellow milk before starting to feed it to the newborn.” [Mother, tribal]

#### Delayed onset of lactation

Women from tribal communities believe that breast milk is the first thing that their children should have, but often compensate for delayed onset of lactation by feeding rice starch and dairy milk. A newborn is fed just enough rice starch and/or milk to quench their thirst: “During the birth of my first baby, it took 3 days for onset of breast milk. Until then, I gave rice starch whenever the child was thirsty” [Mother, tribal]. Mothers also take cues^3^ from the baby to determine the frequency of breastfeeding: “How shall I remember the number of times my baby was breastfed? Whenever the child starts crying, we know that the child should be fed immediately. If the child is silent, it means that she is not hungry at that time” [Mother, tribal].

#### Complementary feeding varies between geographic regions (forest vs. plains)

Once mothers start to lactate, breastfeeding is initiated and continued for a minimum of 6 months to a maximum of 1 year. Breastfeeding is genrally exclusive and depends on the mothers’ understanding. Most mothers in the central zone reported exclusively breastfeeding children at least for a period of 6 months. They started giving water to their children only after 6–7 months, as many feared that children might choke if they were given water at an early age. Mothers from the north and south zones (forest regions) understood exclusive breastfeeding as the absence of any solid food in the daily diet of the baby: “I have never given any solid food other than breast milk to my son until the age of 7 months. However, yes, at the age of 3 months, I started giving him water during the summer season because the child gets thirsty very frequently” [Mother, tribal].

We found evidence of both early (3 to 6 months) as well as late (upto 1 year) initiation of complementary feeding. The most commonly fed items are water, biscuits, bread, fried fritters, rice and pulses. Mothers reported initiating complementary feeding depending on the perceived ability of the children to accept complementary food. “Whenever I am having my meal, I feed her a very little amount of the same food and she got used to it very easily. Then, I realised that I can feed her solid food [and] I gradually started increasing the amount of feed” [Mother, tribal]. Non-tribal mothers start feeding complementary food once the ritual of *‘muh joothi*’^4^ is performed.

#### Rearing practices affect dietary consumption patterns

Children are generally fed by their mothers. When children are able to eat independently, mothers give them food in a separate utensil and leave them to eat with their own hand. Once mothers return to regular work hours, these children are looked after by elders, older siblings, or neighbours. Children are fed up to three meals a day, often consisting of convenience foods, to keep them satiated until the mother returns from work. “Every day before leaving for my work, I leave Rs. 10-20 with my elder daughter to buy something like biscuits or some *pawroti* (breads) from the nearby shops when my younger child gets hungry. This is to keep him going till I get back home in the evening. After that, I feed him breastmilk” [Mother, tribal].

#### Preference for unhealthy, convenience foods

Consumption of convenience foods is common and depends on: (a) affordability, (b) availability of caregivers, (c) onset of breast milk, and (d) mothers’ work. Although all mothers give some amount of foods such as biscuits and packed snacks to their young ones, these foods are fed once in a while. Families that can afford packed snacks usually feed biscuits or similar snacks worth Rs. 5–10 to children once a day. Women reported feeding biscuits after softening them in water to make them palatable for children or using rice starch as a substitute for breastfeeding. “During delayed onset of lactation, the only option for me is to feed my child with rice starch” [Mother, tribal].

Some mothers reported a preference for feeding green leafy vegetables (*katai, saru, munga, jhara, khesari and chana*), since they were perceived to be nutritious for children. These green vegetables are cooked in rice starch and fed along with boiled rice. The community depends on forest produce, namely seasonal leaves, flowers, fruits, and tubers. Potato and dal with boiled rice are the most common foods consumed by households during most months and children are also fed the same diet.

#### All illnesses are not the same

The most common illnesses among children were diarrhoea, cough, cold and malaria. All diarrhoeal episodes do not alarm the mothers. Mothers/caregivers do not provide any special care to children if they pass stool up to 3–4 times a day, waiting instead to see if the situation resolves itself. They start worrying when the child appears weak and the stool begins to emit a foul smell and the frequency increases to 5–6 times a day: “For the very first time, when my son was having loose stools, then his face and eyes started becoming so dull. He became so skinny and his limbs became thin. He was not active while playing and gradually became so lethargic.” [Mother, non-tribal]. If he has a sickness like ‘*chor data/chor dant*’,^5^ then he will start passing stools throughout the day in frequent intervals. If it reaches to 6–7 times a day and has a foul smell too, then we realize that the child is unwell and might be sick” [Mother, tribal]. The other illness of concern is cerebral malaria, which can be fatal.

#### Perceptions of severity determine care seeking behavior

Care seeking behaviour within the community depends on their (a) perception of causality and (b) seriousness of illness episodes. The choice of provider varies depending on the illness and perceived credibility. Home-based care (through local health traditions) is generally practiced for common childhood ailments (fever, cough, cold and diarrhoea).

Fevers or a cough and cold generally do not concern parents/caregivers unless children frequently fall ill or lose appetite. In such cases, the first points of contact are the traditional health practitioners^6^ or faith healers^7^ who determine whether the child is suffering from the effect of an evil eye, fever, or brain malaria, and provide suitable treatment or a *‘tabij’* (amulet) after offering prayers. If a child frequently passes foul smelling stool 6–7 times per day, they identify this as *‘chor data/chor dant’* and seek treatment from traditional healers.

#### Lack of autonomy among mothers

Mothers lack autonomy when it comes to dietary practices, early child care and feeding, and access to health care: **“**I never take any decisions alone about where to take my child for treatment. It is my husband and the in-laws who take decisions” [Mother, tribal]. While dietary practices for pregnant and lactating mothers and child feeding are governed by elders in the family, the decision to access health care and choose a care provider is also influenced by the husband and elders in the community.

#### Limited diet of mothers

Mothers follow a strict diet of non-spicy foods and their diet is limited to plain rice, dal and garlic to protect their infants from cold, indigestion, ulcers, or other infections. “We mothers are not allowed to have spicy food; we usually eat plain rice and *dal* only with one clove of garlic without any spices or vegetables until the child turns 6 months old. We are lactating mothers; if we eat such spicy foods, then the child will be unable to digest the milk and get diseases like mouth ulcer and sometimes boils too” [Mother, tribal]. Certain foods, such as drumsticks, puffed rice, banana and meat, are avoided by mothers until the child attains the age of 12 to 18 months.

The prevailing food practices for pregnant and breastfeeding mothers are not conducive to prevent malnutrition among mothers and children. There are strict norms as to what and how much food a woman can eat during pregnancy. **“**We are not allowed to have food three times a day. We are allowed only one meal a day by our mother-in-laws; [this practice] is supported by our husbands too**”** [Mother, tribal].

#### Understanding malnutrition as “weakness”

The community has a traditional or *emic* understanding of malnutrition as generalized “weakness” among children. They have a set of explanations associated with this condition and seek care depending on the ‘degree of weakness’. Mothers describe weakness as lethargy, inactiveness, wailing, becoming so thin that bones become visible, and inability to move. “I noticed that my child was not that active as my other children in the family. I started observing the difference in my child compared to the children of his age in this village. Unlike other children, he looks inactive, lethargic and even stopped playing. He started getting sick frequently with fever, cough, cold and frequent diarrhea. I ignored it many times thinking that it will be cured by treatment. However, once my child got severe diarrhea, he became extremely weak” [Mother, non-tribal].

Weakness is ‘normalised;’ there is not much concern unless the child reaches an extreme state of weakness or gets sick. “In my family, most of the members are very skinny. My daughter was also born very thin. Even her father is very skinny. So, it is normal for us” [Mother, tribal].

In contrast, mothers having babies who were perceived to be healthy at birth get worried if their child starts becoming thin. “My child was gradually losing his weight and his limbs became so thin. This made me worried and I told my neighbours. One of the neighbours who is a Sahiya in another village told me about the ATFC running in the nearby villages, and to take my child for a checkup” [Mother, tribal].

#### Weakness is caused by sickness or the evil eye

The community distinguishes two possible causes of weakness among children: (a) an outcome of sickness, and (b) evil eye. Mothers are able to correlate episodes of weakness and sickness. They perceive that since their children are weak, they fall ill frequently and become weaker and that such children require improved diet and care: “My child started getting sick very frequently. Every other week, he would fall ill or have a cough and cold. Because of the frequent illness, he started getting weak day by day. And I think that because of the weakness, he again gets sick easily, unlike my other children who get sick very rarely” [Mother, tribal].

If a child looses appetite, wails constantly, or becomes weak and lethargic despite adequate feeding and the absence of visible sickness, then the parents perceive this as an outcome of “evil eye” and seek treatment from faith healers who treat them for “nazar” (evil eye). The community reports that traditional healers often diagnose this later stage of weakness among children as *puni* or *dhena* and appropriate treatment is provided accordingly.

#### Seeking care from qualified doctors in case of extreme weakness

In cases of extreme weakness with manifestations of lethargy, extreme thinness and wrinkles on skin, the families seek medical care from qualified/formal medical professionals in government and private hospitals even if the child is not suffering from any clear symptoms of illness such as fever, cough, cold or diarrhoea. At the same time, families often stay engaged with traditional health practitioners or faith healers. However, there is evidence of increasing awareness that a weak child can be treated at the ATFCs (set up by MSF).

#### Limited access to primary healthcare services

In tribal villages, home visits post-delivery are made by the Sahiya only after Narta.^8^ Mothers reported that they did not receive any counselling or support services for child care and feeding from FHWs during the first week. After *narta*, either the frontline workers come for home visits or the mothers are called to the AWC for recording the child’s weight. Home visits are mostly limited to weighing the baby.

AWWs are responsible for providing daycare and nutrition support. AWWs have limited their activities to supporting Village Health Nutrition Days (VHNDs), recording weight measurements, and distributing supplementary nutrition. All mothers are not informed about the VHND; only those who are listed in the micro-plan for immunization of the child are informed and called by the Sahiyas. Mothers who attend VHND for immunization reported weighing their children on that particular month; the next weighing happens during the next immunization session.

#### Mothers are not informed about weight measurements or changes

Mothers reported that the FHWs do not share weight measurements or MUAC readings. “No, I have never been told about the weight of my child after the VHND. I don’t even know whether or not we are supposed to ask about it” [Mother, tribal]. Mothers reported that FHWs inform them when children are in the ‘yellow or red’ zone, and counsel them to feed their children “well”: “When they measured my daughter’s limbs with the tape, it came in as yellow. Then, I was told to feed her well with whatever is available with us” [Mother, tribal]. Since all mothers whose children are enrolled in ICDS do not visit each VHND, regular screening, early identification and referral of SAM cases for CMAM or facility-based management gets delayed.

When children are identified as SAM or screened for malnutrition, Sahiyas and AWWs counsel the parents to visit the MTC at the *anumandal hospital* (First Referral Unit, Chakardharpur) for treatment. “In the ATFC, I have seen many children are getting cured and looking so healthy. Therefore, I decided to take my daughter to the ATFC for the treatment” [Mother, tribal].

#### Malnutrition as a “new disease”

While mothers of children who have not received care at ATFCs identify a malnourished child as weak, mothers of children who are ATFC beneficiaries identify malnutrition as a form of illness (i.e. *dhena-puni*) occuring among young children (6 months to 2 years). The ATFC beneficiaries perceive *kuposhan* (malnutrition) as a new terminology or disease that has recently become popular. “Earlier, we were not aware of this new disease. In fact, most of us heard this name for the first time” [Mother, tribal]. These mothers believe that malnutrition can be cured by eating the ready-to-use therapeutic food (RUTF) packets provided by MSF.

### Understanding the context: the social determinants

#### Poor food security

Agriculture is the primary activity to secure food as well as income. The lean season lasts for more than 6–8 months, leading to financial difficulties and food shortages [17]. Limited means for income generation result in careful decisions on expenditure and poor dietary intake through the year. The staple diet comprises two meals of rice per day. Green vegetables are consumed only for a few months and dried leafy vegetables and tubers are primarily consumed during the lean season [18]. Consumption of animal protein such as milk, egg and meat is rare [19].

#### Seasonal migration

Seasonal male migration is a common practice among households from the peripheral villages/forest villages as one of the most important coping mechanisms for chronic poverty. Migration takes place in two cycles: post sowing of paddy and post harvest. This compounds the burden of child care, household chores, and income generation on mothers. Women from nuclear households, in particular, make trade-offs between child care and earning a livelihood, which has direct effects on timely child feeding, frequency of feeding, and access to health care. Healthcare, including care seeking for malnutrition, is delayed until the illness nearly turns into a crisis.

#### Limited environmental resources

There is limited access to safe drinking water; shallow handpumps are the only community sources. These are installed by the public health engineering departments and the maintenance is supported through the funds of the Village Water and Sanitation Committee (VWSC). Hardly any households have toilets and open defecation is a common practice. There are no drainage facilities and small pools of waste water can be found in each house where utensils and clothes are washed.

#### Inadequate hygiene practices

Mothers and caregivers do not maintain adequate hygiene for children. Children are not bathed regularly and hand washing before eating or feeding is not a common practice. However, few mothers were particular about washing hands before feeding, especially mothers of children who were ATFC beneficiaries.

### Perceptions and practices by health providers

#### Differing perceptions of malnutrition between state and MSF providers

AWWs and ASHAs/Sahiyas understand malnutrition as *kamzori* (weakness) in a child above the age of 6 months, caused by inadequate dietary intake. Some AWWs attributed malnutrition to the lack of birth spacing. “In my understanding, malnutrition is just weakness. When a child does not get good and healthy foods, it makes them weak gradually” [AWW]. On the other hand, MSF’s Nutrition Link Workers (NLWs) are trained to disseminate health messages that describe malnutrition as a disease. They believe that malnutrition among children can be cured through consumption of take-home rations (THR) and perceive RUTF as a medicine that can cure SAM.

#### Health providers are underutilized

The role of ASHAs is limited to assisting mothers for ante-natal care (ANC), institutional delivery, and post-natal care (PNC). The ASHA/Sahiya is unaware of their role in visiting a mother once her child completes the first 6 months to initiate complementary feeding. An AWW is responsible for visiting newborns and mothers to encourage colostrum feeding, exclusive breastfeeding, and recording the weight of the newborn. None of the mothers reported being visited by AWW in the post-natal period. The activities of AWWs are limited to growth monitoring and distribution of supplementary nutrition. The role of the ANM (most qualified and trained among the three frontline cadres) is limited to providing services only through the VHND sessions: immunization, screening malnutrition and family planning.

#### Inadequate screening and identification of undernutrition

Screening and identification of undernutrition among infants at the household level is conducted by ASHAs till the age of 6 months. They undertake screening under the Integrated Management of Neonatal and Childhood Illnesses (IMNCI) within 3 weeks of birth. They also check the temperature and the weight of the newborn. A baby is identified as ‘weak’ if the temperature increases and weight decreases. For children above the age of 6 months, screening is done by ASHAs and weights are recorded at VHND sessions.

Prior to the MSF intervention, ASHAs used to screen only by weight-for-age (WFA) criteria; this has subsequently changed to mid-upper arm circumference (MUAC) criteria. However, this is not the case in every village; the participation and competence of the ASHAs in screening and identifying undernourished children in the most remote or forested villages is sub-par. Identification of undernourished children at the household level is mostly based on “physical appearance:” very skinny, extremely thin limbs and legs, bulging belly, if mothers inform them that a child’s dietary intake is reducing continuously, or if the child does not enjoy meals.

#### Frontline workers are not confident about the MUAC tape

The ASHAs were not confident in using the MUAC tape. They merely remember the colors of the MUAC tape; red is recognized as the zone where the child is identified as malnourished and yellow and green are both considered normal. Most do not understand the core concepts and the measurements. While entrusted with the primary responsibility of screening, ASHAs generally rely on AWWs and ANMs.

#### Counselling and referral pathways

The AWW weighs children and plots the growth chart at the VHND session with the help of the ASHA. MUAC is used for screening children above 6 months to identify malnutrition. If the child is identified as having moderate acute malnutrition (MAM), then the FHWs (ANM, ASHA, AWW) counsel mothers for supplementary nutrition feeds in the form of THR provided in the AWC and VHND and other nutritious foods such as fruits and vegetables. If they diagnose the condition as severe acute malnutrition (SAM), mothers are advised to report to the ATFCs or MTC (in case of medical complications).

In the MTC, severe illnesses or medical conditions are stabilized by admitting the children and categorising them as ‘Phase I’, ‘Transition’ or ‘Phase II’. Children are categorized as Phase I in cases of medical complications, low appetite, or limited weight gain, and are treated with F75. Children are categorised in the transition phase when medical complications have stabilized or appetite has returned to normal and oedema is significantly reduced. After stabilization of appetite and medical complications, the child is transferred to phase II which entails continuing treatment at the ATFC. In cases of severe medical complications, children are referred to Chaibasa MTC for better treatment and proper medical care. Most of the referrals are through ATFCs and NLWs, often in collaboration with ASHAs. Children with medical conditions like acute diarrhea or severe weight loss may be admitted directly from nearby blocks or villages (based on the advice of ASHAs).

### Continuum-of-care (CoC) processes

#### Gaps at the level of FHWs

There is grossly inadequate support for children discharged from ATFCs/MTCs. The ATFC team provides 1 month’s stock of the RUTF packets after discharge. None of the AWCs provide extra THR to children who are discharged from the ATFC or MTC. The awareness level of AWWs regarding support programs, health care programs, and services for malnourished children are only restricted to the MTC. Most AWWs were not aware about the ATFC and its interventions. “I have heard about the people who are working for treatment of malnourished children. I have not visited the ATFC ever. I have never seen treatment of the malnourished child done at the ATFC” [AWW].

#### Counselling and support services at MTC are not patient centric

Mothers accompanying children who are admitted to the MTC are provided a bed for staying with their child. Most of the time, there are other younger siblings who also accompany mothers to the MTC. Mothers are provided with three meals a day, drinking water, toilet and bathroom facilities, and transportation costs. Lactating mothers are provided with a ration comprised of cereals, pulses, vegetables, meats, eggs or fish once per week. Mothers reported that they did not like the food provided at the MTC. Rice is consumed three times a day by these communities, but the MTC provides wheat-based diets and relatively less rice. This creates dissatisfaction during longer stays. Mothers are individually counselled daily on feeding and weaning practices by the MTC team, which is comprised of on-duty doctors, nurses, and health educators, focusing primarily on nutrition and food intake (with an emphasis on local food items). Mothers are also involved in activities for psychosocial stimulation and counselled to let their children play with family members as well as the toys. However, advice during discharge is limited to nutrition counselling.

#### Poor follow-up by FHWs

Information is shared to the FHWs (generally the ASHA, not other FHWs) when SAM children are discharged from the MTC. MSF provides ASHAs with Rs. Two hundred as incentive support when a SAM child is cured. Follow-ups are therefore not conducted by other FHWs and occasionally skipped by the ASHA despite incentives. “In total, I have been paid at least Rs. 300/- for the identification and admission of identified children to the ATFC. During admission, I have been paid Rs. 100/- and rest Rs. 200/- was given at the time of discharge- that means [after] the child gets cured” [ASHA]. Only ASHAs reported visiting ATFCs when SAM children are recruited to the program.

#### Procedural changes in active screening have created confusion

##### Pre-MSF CMAM

ASHAs conducted active screening at the household level and passive screening at the VHND sessions to identify children with Grade III and Grade IV malnutrition (for referral to the MTC) prior to MSF intervention. The screening tools were WFA and the MUAC tape at the village level.

##### Post-MSF CMAM

After MSF intervention through the CMAM programme, the FHWs conduct screenings with the MUAC tape and identify SAM cases in the village and refer them to the ATFC. At present, only the ASHA conducts screening at the household level. The identified SAM cases are then referred to the ATFCs; however, most ASHAs had not referred SAM children for the last several months.

There is confusion among FHWs between the two methods of identifying malnourished children. Earlier, they were acquainted with the grading system of identification, but with the introduction of the MUAC method, they were confused about how to identify appropriate cases for referrals.

### Barriers and challenges

#### Nutrition link workers find active screening challenging

The NLWs (of the MSF program) screen children in the community using the MUAC tape. Before screening, they first identify children who are physically weak and look very skinny. Other than the physical appearance, children with medical conditions (commonly, fever and diarrhea) are also screened and sent to ATFCs. Oedema is not considered during screening. NLWs find active screening challenging due to the remote location of the forest villages, which often leads to exclusion of cases. The NLWs prefer to conduct active screening in the VHND, where they can identify SAM children and bring them to the ATFC. The FHWs disseminate information to mothers about VHND based on their immunization list. Dependence of NLWs on VHND alone for case identification leads to exclusion of malnourished children who do not attend VHND. Villages that are easier to access in terms of distance and terrain are covered fully by NLWs.

#### Counseling of MAM children is weak

NLWs are required to identify MAM children through active screening and counsel families for locally available nutritious food items. While screening and enlisting is reasonably adequate in most villages, counselling services are inadequate; NLWs recommend locally available nutritious foods, but specific details were observed to be missing. Several NLWs were unsure about services available in the AWC and are not informed whether mothers are receiving services from the AWC or not. The major focus of NLWs is on ensuring that SAM children are enrolled in the programme.

Many of the health educators are not fluent in the local languages (Ho and Odiya) and face major challenges during counselling. They also find it challenging to quickly establish a trusting relationship with mothers and caregivers during the initial visits.

#### Poor coordination between health providers

The NLWs do not seem to be active in their village or community and they have limited interactions with village leaders and FHWs. There is evidence of some tension between other government health workers because of the higher salary paid by MSF for very similar work.

NLWs seem to be less aware of the importance of collaboration with FHWs and their role in the programme. They are only accompanied by ASHAs during home visits for screening on rare occasions. NLWs find it challenging to convince mothers and family members to adhere to the treatment plan and ASHAs or AWWs do not accompany the NLWs during these follow-up visits. The CMAM programme seems to be running in parallel to government institutions without ensuring adequate collaboration and participation of FHWs.

#### Lack of internal coordination within MSF

HEs supervise the NLWs in their respective ATFCs. There are no cogent monitoring mechanisms to review daily activities conducted by NLWs. NLWs from the most remote and forested villages find it difficult to communicate on a daily basis with the HE due to poor network connectivity which hinders feedback and support.

#### Community engagement is inadequate

NLWs are required to participate in village-level meetings with the Munda, Mukhiya and other key persons or active groups to discuss SAM cases and malnutrition. In practice, they hardly ever attend such village meetings as they lack clarity about the need for such participation.

The panchayat (local self-government) members of the village are responsible for ensuring that members of the Village Health & Sanitation Nutrition Committee (VHSNC) are available to support the VHND sessions. PRI members do not visit the AWC on the VHND and are not aware of the need to participate in VHND sessions: “I don’t know whether I am supposed to attend the VHND or not. I have never been asked to be present at the day of VHND” [PRI member].

## Discussion

The nutritional status of a child is not determined by feeding alone; it is an outcome of multiple drivers. The socio-economic position of a household, access to water and sanitation, and access to health care services are some of the risk factors for stunting [20]. This study reveals important gaps in perceptions and practices regarding malnutrition among under-five children in Chakardharpur with respect to food insecurity, poverty, and suboptimal support systems. A household’s capacity to provide adequate nutrition to children depends largely on factors such as geography, limited livelihood opportunities, poverty, food insecurity (especially among forest-dependent tribal households) and multiple migration cycles [21]. The vulnerability is accentuated during lean seasons [22].

Poor feeding and caring practices contribute to childhood malnutrition, which is often aggravated by suboptimal health service coverage [23]. Gaps in the social safety net and welfare schemes (with respect to access and availability of safe drinking water, toilets, food rations, and day care centres) act as key social determinants of malnutrition in the community. Mothers constantly juggle competing demands for time—livelihood, household chores, care of other siblings—which has a bearing on optimal care and feeding of young children, especially those at risk or suffering from malnutrition. This inability to provide appropriate and timely care to children (at risk, sick and malnourished) is often in the context of a normalized experience of malnutrition [19]. Poor health literacy, delay in access to care, time constraints faced by mothers, and community perceptions of malnutrition as a disease inform the ‘decision to seek care’ [24]. The success of the CMAM program is contingent on addressing these drivers alongside providing care and treatment for children who meet screening and referral criteria.

The study points to several critical issues related to strengthening the CMAM program, especially with respect to knowledge and skill gaps of personnel as well as operational needs. The implementation of the CMAM program in its current form entails certain bottlenecks that include a lack of defined responsibilities, lack of clarity in roles, inadequate coordination within MSF, and lack of collaboration between MSF and the existing public health system.

### Strengthening the capacity of health staff

Regular refresher training sessions need to be held for NLWs and HEs to address gaps in service provision. These sessions should clarify the content and execution of counselling sessions with the community, in order to train the HEs and NLWs on how to disseminate positive health care practices and actively engage the mothers.

For HEs, these trainings should cover how to record the social history of ATFC beneficiaries, so that counselling can be tailored. For NLWs, the training should also include regular exposure visits to other ATFCs. They should receive on-site trainings on identifying sick children, MAM cases, and complicated or uncomplicated SAM cases in order to improve active screening, passive screening, and referrals. NLWs must also be trained on the specific care needs of children in these categories and be made aware of the services available for them at the AWC, ATFC, and MTC, so that appropriate care and referral decisions can be made.

The program would also benefit from an expansion of the roles of NLWs and HEs. We observed that NLWs require supportive supervision to conduct field operations (coordination, screening, and referral). The role of HEs needs to be extended to provide active supervision and support to NLWs. Potential areas of engagement include coordinating with NLWs for screening and identifying new cases, and providing technical support and supervision to NLWs during screening, counselling, and follow up.

### Ensuring continuum of care and prevention of relapse

A document registering a detailed social history should be prepared along with the medical history of each beneficiary. This complete document report for each beneficiary must be easily accessible across different levels (AWC, ANM, ATFC and MTC) to facilitate smooth transition and continuum of care. The MIS must include a complete listing of sick MAM children who come repeatedly to the ATFC or MTC, but do not qualify for admissions. These cases can be linked to the AWC/SC/FRU for appropriate care services. Weekly reports for children discharged from the ATFC must be maintained and monitored weekly for a minimum of 2 months in order to prevent relapse and delays in recognition.

Discharged cases should be regularly followed up and provided with need-based care support to prevent relapse. Context-specific individual care and treatment plans should be formulated for all admissions as part of a revised community engagement strategy. It is advisable to assign personnel for developing individual care plans at each care point (ATFC and MTC), and share plans during care transitions across multiple points (i.e. ATFC, MTC and district hospital, along with referrals back to previous levels). Community-based education regarding adequate nutrition during pregnancy must be strengthened as part of the community engagement strategy in order to ensure mothers are eating a healthy and varied diet.

Collaboration between MSF frontline staff and FHWs regarding cases treated at the ATFC (ATFC beneficiaries and SAM children transferred from MTC to ATFC) must be improved. Treatment details of each beneficiary need to be shared before discharge with the respective AWWs/ANMs, including their specific medical care needs, in order to ensure continuum of care.

## Conclusions

Strengthening the capacity of the existing health staff, ensuring continuity in care, improving ongoing community education and engagement efforts to prevent relapses, and making care facilities more patient-friendly are key components of any successful CMAM model.

## Data Availability

The de-identified transcripts that support the findings of this study are available from the corresponding author, Dr. Sakib Burza. However, restrictions apply to the availability of these data, which were used under license for the current study; hence, they are not publicly available. Data are, however, available from the corresponding author upon reasonable request and with the permission of Medecins Sans Frontieres.

## Abbreviations

ANC: Ante Natal Care
ANM: Auxiliary Nursing Midwives
ASHA: Accredited Social Health Activist
ASHA: Accredited Social health Activist
ATFC: Ambulatory Therapeutic Feeding Centre
AWC: Anganwadi Centre
AWW: Anganwadi Worker
CHC: Community Health Centre
CKP: Chakradharpur
CMAM: Community Based Management of Acute Malnutrition
DH: District Hospital
FAO: Food and Agriculture Organization
FHW: Frontline Health Workers
FRUs: First Referral Unit
GNM: General Nursing and Midwifery
HE: Health Educator
ICDS: Integrated Child Development Services
IEC: Information Education Communication
IMNCI: Integrated Management of Childhood Sickness
IYCF: Infant and Young Child Feeding
MPEC: Monthly Per Capita Expenditure
MPI: Multi-dimensional poverty index
MPI: Multi-dimensional Poverty Index
MSF: Médecins Sans Frontières
MTC: Malnutrition Treatment centre
MUAC: Mid Upper Arm Circumference
NFHS: National Family Health Survey
NGD: Natural Group Discussion
NGOs: Non-governmental Organization
NLW: Nutrition Link Workers
NRC: Nutritional Rehabilitation Centre
NTFP: Non Timber Forest Produce
PDS: Public Distribution System
PRI: Panchayati Raj Institution
RUTF: Ready-to-use therapeutic foods
SAM: Severe Acute Malnutrition
SC: Sub Centre
SCM: Severe Chronic Malnutrition
SD: Standard Deviation
SN: Supplementary Nutrition
TFP: Therapeutic Feeding Program
VHND: Village Health and Nutrition Day
VHSNC: Village Health and Sanitation and Nutrition Committee
WAZ: Weight for Age
WHO: World Health Organization

## Footnotes

1 Between 2004 and 05 and 2011–12, the decline in poverty was 8.3%, almost half the decline at the national level (15.3%). Monthly Per Capita Expenditure (MPCE) of the state (as per the National Sample Survey [NSS] data) has been lower than the national average both in nominal and real terms (both in urban and rural areas).

2 *Munda*: The headman of the village under the traditional system of the local governance system, based on the long historical ‘Munda-Manki System’. His post is hereditary. The Munda is a chief among equals and not the superior or overlord.

3 The mothers do not breastfeed their baby at a specific time interval for a specified number of feeds. The practice of breastfeeding depends on number of times a baby asks for milk. The mothers report that when the baby cries, they understand that the baby is hungry and needs to be fed.

4 A ritual of giving grain to a child for the first time, which is performed by the age of 6 months.

5 Child passes stool for more than 6–7 times a day. The stool gives a foul smell, and is watery in nature or changes color.

6 Traditional health practitioner: They use their five senses to diagnose and treat diseases using medicinal plants and animals.

7 Faith healers: They diagnose the ailments with the mantras (chants) and the causes proclaims are due to the possession of evil spirit. They treat or cure the ailments by chants only.

8 A cultural practice conducted within 6 days after delivery. The family takes mother to the village where prayers are offered after which the community believes that the mother becomes pure eligible to meet people. It is believed that this ritual help mothers to produce enough milk for the child.
